# Food Antioxidants and Their Interaction with Human Proteins

**DOI:** 10.3390/antiox12040815

**Published:** 2023-03-27

**Authors:** Olgica Nedić, Ana Penezić, Simeon Minić, Mirjana Radomirović, Milan Nikolić, Tanja Ćirković Veličković, Nikola Gligorijević

**Affiliations:** 1 Institute for the Application of Nuclear Energy, Department for Metabolism, University of Belgrade, Banatska 31b, 11080 Belgrade, Serbia; 2 Center of Excellence for Molecular Food Sciences, Department of Biochemistry, Faculty of Chemistry, University of Belgrade, 11000 Belgrade, Serbia; 3 Serbian Academy of Sciences and Arts, Knez Mihailova 35, 11000 Belgrade, Serbia

**Keywords:** food antioxidants, human proteins, binding, interaction detection

## Abstract

Common to all biological systems and living organisms are molecular interactions, which may lead to specific physiological events. Most often, a cascade of events occurs, establishing an equilibrium between possibly competing and/or synergistic processes. Biochemical pathways that sustain life depend on multiple intrinsic and extrinsic factors contributing to aging and/or diseases. This article deals with food antioxidants and human proteins from the circulation, their interaction, their effect on the structure, properties, and function of antioxidant-bound proteins, and the possible impact of complex formation on antioxidants. An overview of studies examining interactions between individual antioxidant compounds and major blood proteins is presented with findings. Investigating antioxidant/protein interactions at the level of the human organism and determining antioxidant distribution between proteins and involvement in the particular physiological role is a very complex and challenging task. However, by knowing the role of a particular protein in certain pathology or aging, and the effect exerted by a particular antioxidant bound to it, it is possible to recommend specific food intake or resistance to it to improve the condition or slow down the process.

## 1. Food Antioxidants

Antioxidants are substances able to reduce, prevent, or revert the oxidation of other substances. Alternatively, antioxidants are compounds that scavenge reactive oxygen species (ROS) or act to inhibit their production and/or stimulate the antioxidant defense system, thus reducing the cell-damaging effects of ROS [1]. They are abundant in nature, chemically very different, often little soluble in the aqueous system (body), exogenous antioxidants are cleared fast from the body, they are able to exchange electrons and protons, some can be regenerated, some only participate in a redox reaction, while others bind to substrates.

Food antioxidants can exhibit several modes of action in the body. They are (i) direct antioxidants of oxidized substances, (ii) metal ion chelators (thus, inhibitors of Fenton reaction), (iii) cofactors of enzymes involved in antioxidant activity, (iv) activators of transcriptional factors which induce expression of genes involved in antioxidant activity, (v) modulators of signaling pathways, (vi) modulators of secondary and/or tertiary structure, thermal/proteolytic stability and primary function of bound proteins, (vii) participants in other physiological actions [2,3].

Only a few antioxidants, in the form of supplements, have been approved by the responsible authorities for use in human therapy. There is a great interest in synthesizing more potent derivatives or isolating them as natural products with improved absorption/bioavailability/antioxidant properties. However, supplements cannot completely substitute natural sources of antioxidants since a natural variety of derivatives and their possible synergistic or additive effects cannot be achieved in a lab.

The antioxidant potential of foods can be determined by a number of methods: spectrometric, chromatographic, and electrochemical [4]. Several thousand-fold differences in food antioxidant composition were found [5,6,7]. Antioxidant Food Database offers data on the total antioxidant content (TAC) of more than 3100 items consumed by people worldwide [6]. Generally, foods originating from plants have greater TAC than those originating from animals. Foods with the highest antioxidant content are spices, herbs, berries, nuts, fruits, vegetables, chocolate, and coffee. Dairy products, meat, and eggs have very low antioxidant content. High TAC values, however, are not necessarily proportional to the bioavailability of antioxidants.

## 2. Food Antioxidants, Their Stability, and Availability under Physiological Conditions

Bioavailability is a term that defines the amount of a nutrient that is digested in the gastrointestinal tract, absorbed in the intestine, and further metabolized in the body. This path depends on many factors, such as a chemical form or antioxidant derivative, food matrix, degree and type of transformation by host intestinal enzymes and microbial enzymes in colon microbiota, interaction with other molecules in the body, and transport [8]. Food-derived antioxidants in the body are often not in the same form as in food.

Antioxidants soluble in water can be directly absorbed in enterocytes by diffusion and/or via transporters and further translocated to blood. Lipophilic antioxidants, after digestion in the stomach, form micelles with exogenous lipids and host bile acids released from the gallbladder and further undergo diffusion through the epithelial surface of the intestine. Absorbed micelles are transported as lipoprotein particles via lymph [9]. Modification reactions, which increase their aqueous solubility (most often conjugation), occur during their passage through the liver. Metabolic processes in the liver depend on the nature of the compound. After its physiological life has expired, an antioxidant derivative is directed to excretion via either the kidneys (urine) or the liver (bile/feces). Antioxidants not absorbed in the intestine pass to the colon, and microbial enzymes further process them. Hydrolysis, reduction, decarboxylation, dehydroxylation, and demethylation processes occur, resulting in new derivatives [10,11].

The food matrix plays a significant role in antioxidant availability. Unless free, antioxidants can be covalently bound to macromolecules (e.g., polysaccharides), associated with other molecules via ionic bonds, mechanically entrapped in a food matrix, or physically associated with specific cell structures [12]. The same antioxidant may be free in one food and bound in another. A combination of foods ingested at the same time, due to their mutual interaction, may affect the bioavailability of their components. However, the data on specific food combinations are conflicting. For example, a study by Dupas et al. [13] has shown that, although ingestion of coffee with milk can induce binding up to 40% of coffee chlorogenic acid (CGA) to milk proteins, no significant overall reduction in beverage antioxidant power was noted, most likely due to antioxidant liberation during gastric and intestinal digestion. On the other hand, a study by Duarte and Farah [14] reported that consuming milk with coffee reduces the bioavailability of coffee polyphenols, as assessed by measuring urinary CGA. In general, co-ingestion of antioxidants and proteins may induce their interaction, sometimes resulting in reduced bioavailability of antioxidants (or, rarely, increased). Dairy products, for example, can increase the availability of polyphenols from berries via polyphenol/protein interaction [15].

Contrary to that, co-ingestion of lipids may increase the bioavailability of lipophilic antioxidants, such as carotenoids, by facilitating their emulsification and absorption [16]. The interaction of saccharides and antioxidants may have both positive and negative consequences. For example, sweet wine sugars interact and increase the stability of polyphenols [17]. Alternative sweeteners, such as sucralose and stevia, improve the bioavailability of anthocyanins in beverages [18]. The effect of combining different food ingredients in the simulated gastrointestinal digestion on TAC of combined foods is given for the most commonly consumed foods in the study of Comert and Gokmen [19]. The bioavailability of a particular antioxidant is antioxidant-, matrix- and food processing-specific (such as cooking and fermentation). Examination of TAC in 54 types of beverages prepared from coffee beans revealed that the range of TAC values is 0.89 to 16.33 mmol/100 g and depends on the preparation route [6].

## 3. Binding Characteristics of Proteins and Their Interaction with Food Antioxidants

A proper translation of mRNA, followed by the appropriate post-translational modifications, is essential for adequate protein folding, targeting, and specificity. Proteins are not stable molecules existing as just one well-defined structure. Usually, a population of several conformational states exists, although not necessarily at the same time and location. Protein conformers originate from molecular motions that do not require considerable energy input, giving rise to molecular states of similar stability [20]. A dynamic equilibrium will change in the presence of ligands, which will favor the existence of certain protein conformer(s). If more ligands are present in the surrounding at the same moment, their competition or synergism will further govern the redistribution of the conformer pattern. Based on the concept of the pre-existing population of protein conformers [21], the ligand may be expected to choose the most suitable for the interaction. Ligand/protein binding additionally stabilizes the complex through altered protein folding and establishing new interactions inside the protein and with the ligand. Greater ligand-binding capacities possess proteins with a larger number of conformers, and the same binding site may accommodate even dissimilar ligands. Proteins that can convert to a greater number of conformers exploit more interfaces for interactions [22]. Altered post-translational modifications of proteins as well as ligand binding, may have physiological consequences, affecting protein conformation, stability, activity, specificity, transportation, proteolytic susceptibility, and clearance rate.

Food antioxidants can interact with proteins at several possible places: in a deep protein cavity, where accessibility of environmental solvent is low, in a cleft between protein units, at the shallow surface groove, or at the protein surface where charged amino acid residues are exposed. Bonds that form between the antioxidant molecule and protein are usually multiple, involving many amino acid residues, and mostly non-covalent: hydrogen bonds (H-bonds), electrostatic bonds (salt bridges), hydrophobic interactions, Van der Waals bonds, and π-π interactions. Amino acids that interact with a ligand can be polar or non-polar and belong to the α-helix, β-sheet, or loop region of the molecule. Sometimes, all structural elements are involved. Some interactions are weak, whereas others are strong. Structural adjustments of the protein to accommodate a ligand can affect its conformation, stability, and behavior.

Covalent (irreversible) interactions require specific conditions and/or the involvement of specific reactive groups, which can chemically modify amino acid residues, enabling covalent binding. Such interaction in the case of human blood proteins is a rare event. An example of a covalent partnership is an interaction between epigallocatechin gallate (EGCG) and human serum albumin (HSA). Protein carbonylation occurs when these two molecules are incubated at pH 7.4 and 37 °C. EGCG oxidizes lysine residues at the EGCG-binding sites in HSA, generating an oxidatively deaminated product [23]. EGCG can also form a covalent adduct with glyceraldehyde-3-phosphate dehydrogenase (GAPDH) via the oxidation of cysteine thiol residues in the protein [24]. Oxidized [^14^C]quercetin was shown to bind highly selectively to HSA in vitro covalently [25,26], and such a reaction may be expected in vivo upon releasing hydrogen peroxide/peroxidases from cells.

The effect of antioxidant binding to the protein (if there is an effect) can be activating, inhibiting, or modulating, which depends entirely on the nature of the binding couple. The effect of one antioxidant is usually of the same type on the number of interacting proteins. There are, however, antioxidants that can exert all three categories of effects depending on the particular binding protein. Resveratrol is an example of an antioxidant that can interact with numerous proteins that do not share primary sequence homology or common fold or have other similarities, exploiting all protein known binding places, forming all known types of bonds, with acidic, basic, or neutral amino acids and exerting no common effect [27]. Due to this diversity of behavior, resveratrol was termed a promiscuous molecule. Interestingly, if one protein performs several physiological roles, antioxidant binding may affect only some and not all of them, as in the case of resveratrol binding to estrogen receptor-α, modulating the inflammatory process but not cell proliferation [28]. Resveratrol binding to the receptor causes a conformational change of the protein, enabling the regulation of co-regulatory molecules, which participate in the transcription control. Likewise, a protein can bind different antioxidants at different sites. For example, β-lactoglobulin (BLG) binds apigenin in the internal cavity, luteolin at the entrance of the cavity, and eriodictyol outside the cavity [29].

One antioxidant molecule most often interacts with one molecule of protein. Sometimes, there are more binding places, for example, at more subunits. In addition, there may be several different binding places for the same antioxidant on one protein, as in the case of S-adenosylmethionine synthetase 2, which can bind two resveratrol molecules, one at the substrate binding place and the other at the interface between monomer units [30]. Protein interaction with one ligand may create new binding sites for another type of ligand or cause conformational change, enabling the accommodation of two instead of one ligand of the second type. For example, fatty acids bound to albumin allow the binding of additional resveratrol molecules by creating a more hydrophobic environment [31]. Some antioxidants are present in nature as both *cis* and *trans* isomers, and their interaction with proteins can differ. Additionally, some antioxidants may exist as optical isomers—enantiomers (*R* and *S*). *R*, a naturally occurring form of α-lipoic acid (LA) in food, but not *S*, is an essential cofactor of enzymes involved in oxidative metabolism in mitochondria [32]. A mixture is present in supplements, and it may be expected that optical isomers possibly influence the interaction with binding proteins as well. A protein can preferentially bind one, especially if the interaction stabilizes protein or antioxidant structure. Generally speaking, the uptake, binding, and recycling of an antioxidant are related to the form of antioxidant present, protein, and cell type, as well as the redox status of both the cellular and extracellular environment.

Besides common features of antioxidant activity, particular behavior of antioxidants may be characteristic just for some. For example, both LA and its reduced form, dihydro-LA (DHLA), act as antioxidants and, unlike other antioxidants, are effective in both hydrophilic (e.g., plasma, cytosol) and lipophilic (plasma membrane) environments [33]. LA/DHLA couple was termed a universal antioxidant since it can regenerate other antioxidants, i.e., revert them from oxidized into the reduced form [3]. When their concentration, however, exceeds a certain level (above 75 µM), they behave as pro-oxidants (most likely at the transcriptional level). Such characteristics should be considered when recommending specific food quantities (or supplements).

## 4. Methods for Studying Antioxidant/Protein Interactions

### 4.1. Binding Studies

Investigation of protein fluorescence is one of the most commonly applied approaches to studying the binding of antioxidants. Protein intrinsic fluorescence arises from tryptophan and tyrosine side chains upon excitation at 280 nm. Ligand binding can alter the environment of aromatic side chains, causing changes in the fluorescence emission intensity (usually quenching) and a shift in the position of fluorescence maximum [34]. Thus, examining the intrinsic fluorescence spectra of proteins makes it possible to detect and characterize the binding effect. Titration of protein solution at a fixed concentration by different ligand concentrations, followed by the measurement of fluorescence intensity, is employed to calculate the affinity constant (*K*a) and determine the mechanism of quenching (static or dynamic). Thermodynamic parameters of the interaction, such as enthalpy change (ΔH), entropy change (ΔS), and Gibbs free energy (ΔG), can be calculated by varying the experimental temperature. Negative values of ΔH and ΔG indicate a spontaneous process, as in the case of resveratrol/collagen binding [35]. Although affinity constants range between 10^4^ and 10^8^ M^−1^, the protein fluorescence quenching approach can be reliably employed to determine constants between 10^4^ and 10^6^ M^−1^. An additional method to detect binding relies on the ability of an antioxidant to fluoresce, as in the case of resveratrol and phycocyanobilin (PCB), after excitation at 320 or 460 nm, respectively. The interaction of such antioxidants with proteins induces a conformational change, resulting in altered emission spectra [36,37].

Although fluorescence spectroscopy is widely used to characterize antioxidant/protein interaction, isothermal titration calorimetry (ITC) is a gold standard for the determination of thermodynamic parameters of ligand binding. The procedure assumes the addition of an antioxidant to a protein solution in a calorimeter cell held under isothermal conditions. The method sensitively measures enthalpy change induced by ligand binding [38]. ITC is the only technique that simultaneously determines all binding parameters: *K*a, ΔG, ΔH, ΔS, and stoichiometry in a single experiment. However, the limited aqueous solubility of antioxidants imposes certain limitations and challenges for ITC experiments. For example, adding EGCG to the HSA solution results in enthalpy change originating not only from the binding event but also from the dissociation of initial EGCG aggregates upon dilution [39]. Since antioxidant solubilization often requires the presence of organic solvents (such as methanol or dimethyl sulfoxide), signal artifacts can occur. If an organic solvent is inevitable, a protein solution must also contain the same percentage of that solvent to avoid a signal arising from heat change due to organic solvent dilution [40].

Another technique used for binding studies is surface plasmon resonance (SPR). Although it is usually employed to examine protein/protein interactions, ligand/protein associations can also be detected [41]. The procedure is based on protein immobilization on a gold chip and its exposition to ligand-containing solution overflow. An increase in surface mass due to the formation of a complex is detected as a change in the refractive index of the light beam hitting the surface, which is proportional to the amount of bound ligand. A limitation of this method is the size of the ligand, as it has to be of a sufficient mass to be detectable upon binding. Another limitation is the necessity of recovering the chip under harsh conditions, especially in the case of strong interactions, restricting the number of cycles of the immobilized protein preparation.

Mass spectrometry (MS) can also detect ligand/protein interactions, providing that they are sufficiently strong to withstand vacuum and ionization conditions. Hydrogen bonds, electrostatic interactions, and Van der Waals forces are strengthened or unchanged in a vacuum, whereas hydrophobic interactions are weakened. Several modifications are usually required for the ligand/protein complex to remain stable [42]. This technique is most often used to confirm stable covalent modifications of proteins, as was shown in the case of PCB binding to BLG [43] and BLG glycoconjugate formation initiated by high-intensity-ultrasound-induced Maillard reaction [44].

### 4.2. Structural Aspects

UV/VIS absorption spectroscopy is one of the most straightforward techniques for studying protein conformational change and detecting antioxidant/protein complex formation. The method captures the effects of the environmental change close to aromatic side chains. Another technique, near-UV circular dichroism (CD) spectroscopy, reveals changes occurring specifically in the chiral environment around aromatic structures (protein tertiary structure). Since a protein binding pocket usually contains aromatic moieties, the near-UV CD spectrum sensitively monitors ligand binding [45]. Limitations of the mentioned methods include requiring a relatively large amount of protein (at least 1 mg) for reliable measurements.

Far-UV CD spectroscopy is commonly used to evaluate the effects of antioxidant binding on protein secondary structure. Since experiments require a smaller amount of protein (approximately 0.1 mg), this type of analysis is more frequently employed than near-UV CD measurements. Several software packages have been developed to quantify secondary protein structures, such as α-helix, β-sheet, β-turn, and random coil, facilitating data management [46]. Secondary protein structures are, however, less sensitive to changes caused by ligand binding than tertiary protein structures, limiting the sensitivity of the method. Additionally, measurements in the far-UV region (180–260 nm) may be influenced by buffers and organic solvents, so their appropriate choice is required to avoid unspecific spectral signals [47].

Fourier-transform infrared spectroscopy (FTIR) is an alternative technique for studying the secondary structures of proteins. Unlike far-UV CD, FTIR is not sensitive to light scattering, providing a valuable tool for protein aggregation examination. Several bands characterize the IR spectra of proteins, but an amide I band, arising from C=O stretching frequency, is the most convenient for evaluating [48]. A limitation of this method is its sensitivity to strong absorption of H_2_O molecules in the amide I region, thus requesting signal correction for the buffer contribution. Conduction of experiments in D_2_O would make data treatment much easier since D_2_O does not absorb in this spectral region. However, one should keep in mind that due to the H-D exchange, protein secondary structures may be altered by the replacement of H_2_O by D_2_O. If ligand binding provokes discrete changes in the secondary structure of the protein, employment of both FTIR and far-UV CD is suggested to confirm the effect, as was performed to define an increase of 5% of α-helical structures upon PCB binding to HSA [49].

The formation of the antioxidant/protein complex may also be traced via the absorption and CD spectra of an antioxidant. For example, PCB binding to HSA induces stereoselective interaction only with the M conformer of the ligand, as can be observed by the negative Cotton effect in the CD spectrum of the complex. Furthermore, increased absorbance of PCB in the presence of HSA indicates the transition of this tetrapyrrole from cyclic to more extended conformation upon binding [37]. Increased intensity of the absorption maximum of resveratrol after its binding to fibrinogen indicates its increased solubility [36]. Many antioxidants have low solubility in water, and binding to proteins under physiological conditions increases their concentration in blood [31] and, consequently, prolongs their physiological potential. In that sense, proteins that can interact with numerous antioxidants, such as albumin, and antioxidants that can bind to many proteins, such as resveratrol, probably exert greater therapeutic efficiency.

Small-angle X-ray and neutron scattering methods (SAXS and SANS) provide global information on protein size, shape, and folding state in the solution. Software packages for the analysis of SAXS and SANS data enable the determination of the 3D structure of proteins alone and in complexes with ligands, although at a much lower resolution than crystallographic methods [50,51]. Ligand binding, which induces a conformational change of the protein, influences its flexibility and size detectable by the mentioned techniques. Both SAXS and SANS experiments require relatively high protein concentration (usually a few g/L), which further requests high ligand concentration, imposing the limitation of the approach in the case of ligands poorly soluble in an aqueous medium. The stabilization of BLG with retinol and resveratrol under high pressure was analyzed using this technique [52].

The formation of the antioxidant/protein complex can alter the thermal and/or proteolytic stability of the protein (as a consequence of the conformational modification). Ligand binding can change the melting temperature (Tm) of the protein as well as the shape of the denaturation curve. There are, however, interesting cases when the trend of the melting curve of the complex is different from the trend of the curve originating from the native protein, yet the melting temperatures may be the same. Such an example can be seen in the case of PCB binding to catalase [53]. Increased Tm indicates a more rigid protein structure, which may be more resistant to proteolysis, as in the case of bilirubin and PCB binding to HSA [49,54]. Structurally modified proteins can exhibit greater or lower susceptibility to proteolysis, leading to either shorter half-life and insufficient duration of its function or longer half-life and (undesirably) prolonged activity. Considerably modified proteins may even tend to fragment or aggregate, imposing a risk of their precipitation or formation of new antigens that stimulate the immune system. Certain antioxidant/protein combinations, such as EGCG/trypsin, form aggregates detectable by SAXS and atomic force microscopy (AFM) [55].

**Figure 1:**
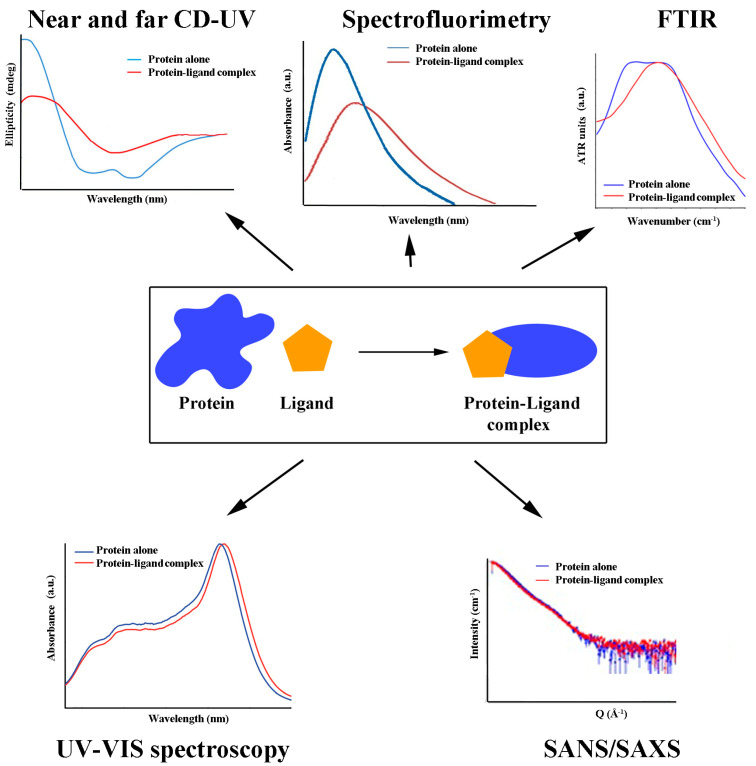
Structural consequences of antioxidant/protein binding.

Docking simulations predict antioxidant binding site(s) to a particular protein by matching known structural data on binding partners and processing them through appropriate software. Out of many produced ligand/protein complexes in a model system, the best docking orientation, the number of ligand/protein interactions, and the docking energy score nominate the most probable binding site and conformation of the ligand. The competition between different antioxidants for the same binding site can also be studied using docking simulations [49] and confirmed further in the so-called displacement experiments. The binding site of DHLA to HSA, for example, was determined in such an experiment, as DHLA could displace previously bound warfarin [56]. Similarly, PCB displaced bilirubin from its binding site at HSA [37]. Both antioxidant competition and synergism (regarding other antioxidants or another kind of ligand) should be considered, as either reduced or increased activity than expected may result under physiological conditions. A general overview of the most frequent findings derived from antioxidant/protein binding is given in Figure 1.

### 4.3. Functional Aspects

Each protein inside the body performs its specific roles, which may be affected by ligand binding. When the ligand is an antioxidant, three aspects are to be considered—the primary functions of the protein, a possible antioxidant effect of the ligand on the protein, and the reducing power of the complex compared to protein or antioxidant alone. Primary roles of the protein can be investigated via functional tests that simulate specific physiological events, for example, enzyme activity, antigen/antibody interaction, receptor binding, cell internalization, cell signaling, coagulation, interaction with other ligands or metal ions, etc. Accordingly, an array of methods is employed for such examination. In vitro assays with cells confirm the biological effects of antioxidant/protein complexes [57]. However, one should bear in mind that not all functions of one protein are necessarily affected by antioxidant binding. Additionally, the binding of one antioxidant may cause effects, but the binding of another may have no effect. Resveratrol binding to fibrinogen, for example, exerts no effect on the hemostatic properties of fibrinogen, including the formation of fibrin and fibrinolysis [58]. In contrast, fibrinogen with bound DHLA forms thicker fibrin fibers, affecting coagulation [59]. PCB binding to catalase does not affect its enzyme activity [49]; the binding of ellagic acid increases the activity [60], whereas resorcinarenes and quercetin reduce the activity [61,62]. Long-chain fatty acids bound to albumin disrupt the binding of zinc ions, while the addition of LA (also a fatty acid) can restore this function [63].

The binding of polyphenols to protein receptors on immune cells can exert an immunomodulatory effect [64]. For example, EGCG interacts with 67 kDa laminin receptor (67LR), zeta chain-associated 70 kDa protein (ZAP-70), and retinoic acid-inducible gene (RIG-I)-like receptors. This antioxidant also exhibits anti-cancer effects by binding to and altering the function of many human proteins involved in cancer onset and progression [65]. EGCG covalently bound to HSA acquires antigenic features and reacts with the natural IgM antibodies [23], while covalently bound to GAPDH inhibits enzyme activity [24]. Naringin binds to the aromatic hydrocarbon receptor (AhR) and induces the production of regulatory T-cells. Baicalin can bind to Toll-like receptor (TLR) 4, T cell receptor (TCR) αβ, and IgM- (sIgM-) B-cell receptor, inducing their up-regulation and influencing both adaptive and innate immunity [64]. If antioxidant binding interferes with a specific protein function, it may be postulated that the binding site is close to or even includes amino acids involved in the protein function. Docking simulations can confirm the proximity or overlapping of sites.

Since all proteins are prone to oxidation, antioxidant binding may affect this susceptibility. One should bear in mind that one protein (e.g., albumin) can be oxidatively modified in many ways, at different sites, by different oxidizing agents [66]. Thus, a particular modification and consequential conformational change may be sensitive to interaction with specific antioxidants. A number of tests can be employed to investigate a protective effect of an antioxidant bound to the protein under artificially induced oxidative stress. Various agents are used for in vitro experiments to generate free radicals, such as 2,2′-azobis(2-amidinopropane) hydrochloride (AAPH), a combination of ferric-chloride and ascorbate, hypochlorous acid, chloramine-T [66].

**Figure 2:**
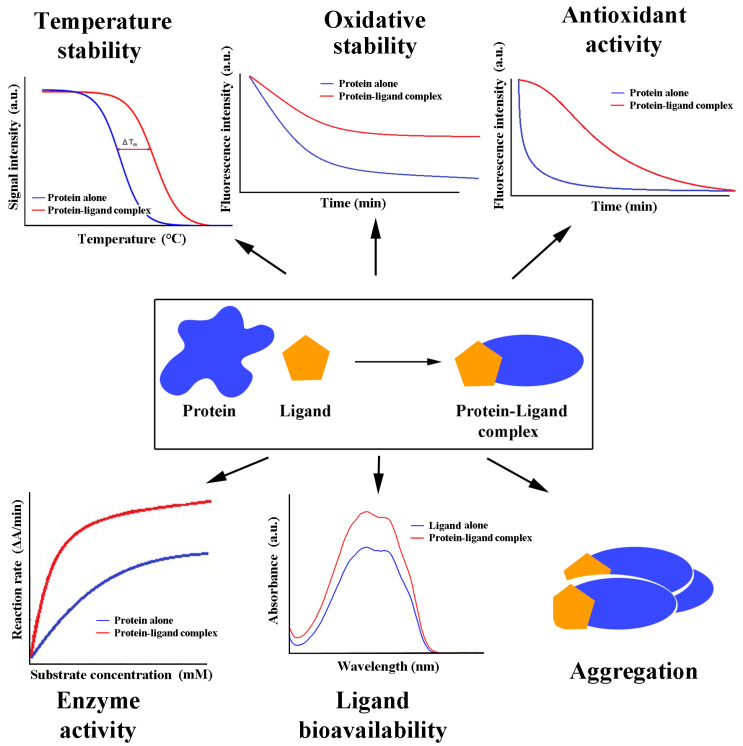
Functional consequences of antioxidant/protein binding.

A decay curve of the native protein molecule results from the reduction in its intrinsic fluorescence with time exposed to a free radical inducer. An antioxidant bound to protein may exert either no protective effect, which can be monitored as a steep decay curve, the same as for the native protein submitted to free radical oxidation, or exhibit some degree of protection, resulting in a smoother decay (oxidation) process. General examples of the effects are shown in Figure 2. Protein protection against oxidation contributes to proper and more prolonged protein function. Furthermore, antioxidants bound to protein may reach targets, sites, and compartments where they would not be found in a free form. Finally, when a protein is directly involved in a process that includes redox events (e.g., catalase), the bound antioxidant may contribute to the final effect. Experiments for determining the reducing power (antioxidant activity) of the protein alone and in the complex with an antioxidant have shown that proteins in complexes may have unchanged, decreased, or even increased reducing power if the protein alone exhibits almost no such effect [36].

Not only protein but an antioxidant itself can be oxidatively stabilized in a ligand/protein complex, increasing its bioactive potential. The effect originates from the antioxidative activity of the protein. Examples of such proteins are phycocyanin and BSA [67,68]. Many research articles, however, report on the reduction in the antioxidant activity of polyphenols in the presence of proteins, as assessed by free radical scavenging methods. The plasma’s antioxidant capacity may increase after adding an antioxidant (e.g., quercetin, rutin, and catechin), but the enhancement is smaller than the antioxidant capacity of an antioxidant itself, suggesting partial masking of this feature [69]. The masking effect on quercetin activity examined in deproteinated plasma was smaller than in native plasma, confirming that its interaction with proteins was influential [70]. Trypsin proteolysis of quercetin/BSA complexes demonstrated an increase in the total antioxidant activity of the degradation fragments, although not reaching the activity of an equivalent amount of free quercetin [71]. Likewise, binding quercetin, luteolin, kaempferol, or apigenin to trypsin reduces the proteolytic activity of trypsin and the radical scavenging activity of flavonoids [72]. The quantitative effect depends on the number and position of hydroxyl groups in flavonoids.

The knowledge collected by examining interactions discussed so far was further applied to form complexes on purpose with additional covalent bonds, providing more stable associations that affect protein significantly. Since this article intends to review interactions that can occur in circulation, artificial modifications will be briefly mentioned. Only certain amino acid residues can form covalent bonds but can also be chemically modified to introduce new reactive groups [73,74,75]. Covalent modifications of proteins represent a significant area of research in food chemistry, as ligand binding can lead to the creation of proteins with desired techno-functional properties, improved digestibility, or reduced allergenicity. Examples of such modifications are PCB/BLG complexes [76] and laccase cross-linked BLG [77,78].

For the purpose of this review article, interactions between antioxidants classified according to their chemical structure and major human proteins in circulation, together with the observed effects, were examined in the literature. SCOPUS database was used to collect information for the period 2000–2023 (February). Major proteins were defined as those whose referent concentration in the blood reaches two g/L or more. Data on plasma proteins: albumin (38–51 g/L), immunoglobulin (Ig) G (8–18 g/L), IgA (0.9–4.5 g/L), IgM (0.6–2.5 g/L), transferrin (1.8–3.8 g/L), α2–macroglobulin (2–4 g/L), and fibrinogen (2–4 g/L), as well as whole blood protein hemoglobin (115–170 g/L), were searched for. No results were found for the interaction between immunoglobulin molecules and any antioxidants. As for other antioxidant/protein interactions, only data for proteins that could be undoubtedly identified as human were included in this article. Known interactions between food antioxidants and chosen human proteins (albumin, transferrin, α2-macroglobulin, fibrinogen, and hemoglobin) are listed in Table 1, Table 2, Table 3, Table 4 and Table 5 (each table for one protein).

The most frequently studied interactions are between antioxidant molecules and HSA (Table 1). Interactions with 86 antioxidant species were recorded. Less work was carried out with other proteins; only a few research groups investigated those (Table 2, Table 3, Table 4 and Table 5). Most papers report only the existence of the interaction, most often evaluated via structural changes of proteins. A few articles describe the functional effects of complex formation on either protein or antioxidant molecules. Data on *K*a values for the same interacting pair vary between studies, most likely due to different experimental conditions applied. Therefore, the research field on antioxidant/protein interactions remains widely open.

Some data found during the literature search were conflicting. For example, Zhang and coauthors reported an increase in the α-helix content of HSA upon interaction with gallic acid [79], whereas Chanphai and Tajmir-Riahi [80] reported its reduction. Only articles on the direct interaction between one antioxidant and one protein were considered for this review. There are, however, studies that examine the binding of whole plant extracts to human proteins. For example, extracts containing polyphenols and monoterpenes from berries interact with HSA with different binding affinities related to their antioxidant properties [81]. At the same time, *Cassia fistula* leaf extract-based gold nanoparticles were shown to interact with HSA inducing minor changes in its structure [82]. Effects of these interactions could not be attributed to a single binding couple and were, thus, not included in tables.

Bovine, porcine, or sheep protein interactions with antioxidants were also examined. Sometimes there are only data for a particular antioxidant and animal protein complex. For example, the binding of thymol [83], limonene [84], and lycopene [85] was studied using only bovine serum albumin (BSA). Due to the high degree of homology between animal and human proteins, authors usually suggest that data obtained with animal proteins could be extrapolated to human proteins. This is, however, not always the case, as was documented for PCB binding to HSA and BSA [37,67]. Different PCB conformers bind to HSA and BSA. Although two PCB binding sites exist on both proteins, one is the same in HSA and BSA, whereas the other is not. Similarly, the binding of scopoletin to HSA and BSA, although with a similar effect on protein secondary structure, is governed by different forces. Scopoletin/BSA complex is mainly established via Van der Waals and hydrogen bonds, while complex formation with HSA involves hydrophobic and electrostatic interactions [86]. Taken together, results that characterize interactions of antioxidants with proteins from different species should be treated as species-specific.

**Table 1:** Known interactions between food antioxidants and human albumin.

Antioxidant Class (Subclass)	Chemical Compound	Effects (the Binding Constant in M^−1^, if Available) [Reference]
Flavonoids (Flavonols)	Quercetin	No significant influence on the structure [87]; no effects were studied [88]; inhibition of the oxidation of HSA-bound linoleic acid (1.2 × 10^5^ at 25 °C) [89]
	Fisetin	No effects were studied (1.2 × 10^5^ at 25 °C) [88]
	Galangin	No effects were studied (2.3 × 10^5^ at 25 °C) [88]
	Rhamnetin	No effects were studied (1.3 × 10^5^ at 25 °C) [88]
	Myricetin	No effects were studied [90]
	Kaempferol	HSA unfolding to some degree (3.5 × 10^5^ at 25 °C) [91]; negligible structural alteration (3.5 × 10^5^ at 25 °C) [92]
	Morin	Reduction of α-helix and β-sheet structures (1.1 × 10^5^ at 37 °C) [93]
	Astilbin	Reduction of α-helix content of HSA and antioxidant capacity of astilbin (4.5 × 10^5^ at 37 °C) [94]
Flavonoids (Flavones)	Luteolin	Reduction of α-helix and increase in β-turn structures; altered configuration of two disulfide bridges (1.6 × 10^5^ at 25 °C) [95]; HSA stabilization by inhibition of fibrillation and glycation [96]
	Apigenin	No effects were studied (1.3 × 10^5^ at 25 °C [88]; 4.6 × 10^6^ at 20 °C [97])
	Rutin	No effects were studied (0.7 × 10^5^ at 25 °C) [98]; reduction in α-helix content (2.4 × 10^6^ at 25 °C) [99]
	Chrysin	No effects were studied (2.0 × 10^5^ at 25 °C [88]; 2.5 × 10^5^ at 25 °C [100]); stabilization of HSA by inhibition of fibrillation and glycation [96]
	Diosmetin	No effects were studied [88]; slight alteration of HSA structure (1.2 × 10^5^ at 25 °C) [101]
	Flavone	No effects were studied (0.6 × 10^5^ at 25 °C) [88]
	Trimethoxy flavone	Partial unfolding of protein secondary structure (1.0 × 10^3^ at 25 °C) [102]
Flavonoids (Isoflavones)	Genistein	Binding of oleic acid decreases affinity to HSA (7.8 × 10^6^ at 20 °C) [103]; reduction in α-helix content (0.2 × 10^5^ at 25 °C) [104]; no effects were studied (0.5 × 10^5^ at 25 °C [88]; 1.5 × 10^5^ at 27 °C [105]);
	Formononetin	No effects were studied (0.2 × 10^5^ at 25 °C) [88]; alteration of HSA structure (0.6 × 10^5^ at 25 °C) [106]
	Daidzein	No effects were studied [105]; slight alteration of HSA structure (7.8 × 10^6^ at 20 °C) [107]
	Prunetin	No effects were studied (0.4 × 10^5^ at 25 °C) [88]
	Biochanin	Reduction of α-helix content (0.2 × 10^5^ at 20 °C) [108]
Flavonoids (Flavanols)	Catechin	Reduction of α-helix content (2.9 × 10^5^ at 25 °C [80]; 0.2 × 10^5^ at 20 °C [109])
	Epicatechin	Prolonged stabilization of ligand [110]
	Epigallocatechin	Prolonged stabilization of ligand [110]
	Epicatechin gallate	Prolonged stabilization of ligand [110]; reduction in α-helix content (3.1 × 10^5^ at 25 °C) [80]
	Epigallocatechin galate	Protection and stabilization of ligand from oxidation [111]; prolonged stabilization of ligand [110]; reduction in α-helix content (3.3 × 10^5^ at 25 °C [80]; 3.2 × 10^5^ at 25 °C [112]); increase in protein aggregation and promotion of heterogeneous aggregate formation [113]
Flavonoids (Flavanonols)	Taxifolin	Alteration of HSA conformation (1.8 × 10^5^ at 37 °C) [114]; reduction in α-helix content (1.1 × 10^5^ at 25 °C) [115]
Flavonoids (Flavanones)	Hesperetin	Reduction of α-helix content (0.2 × 10^5^ at 25 °C [116]; 0.8 × 10^5^ at 25 °C [117])
	Narirutin	No effects were studied (0.7 × 10^5^ at 27 °C) [118]
	Naringin	No effects were studied (0.3 × 10^5^ at 27 °C) [118]
	Nobiletin	No effects were studied (1.3 × 10^5^ at 27 °C) [118]
	Tangeretin	No effects were studied (1.0 × 10^5^ at 27 °C) [118]
	Naringenin	The binding of oleic acid decreases its affinity to HSA (5.3 × 10^6^ at 20 °C) [103]; no effects were studied (0.7 × 10^5^ at 27 °C) [118]
	Flavanone	No effects were studied (0.5 × 10^5^ at 25 °C) [88]
	Sakuranetin	No effects were studied (0.2 × 10^5^ at 25 °C) [88]
Flavonoids (Anthocyanins)	Pelargonidin	Lower pH induces stronger binding (2.1 × 10^5^ at 37 °C) [119]
	Cyanidin	Lower pH induces stronger binding (3.1 × 10^5^ at 37 °C) [119]
	Delphinidin	Lower pH induces stronger binding (3.4 × 10^5^ at 37 °C) [119]
	Malvidin	Lower pH induces stronger binding (1.7 × 10^5^ at 37 °C) [119]
	Pelargonidin-3-*O*-glucoside	Lower pH induces stronger binding (3.7 × 10^5^ at 37 °C) [119]
Flavonoids (Dihydrochalcones)	Phloretin	Reduction of α-helix content; increased resistance to aggregation, fibrillation, and oxidative modification (5.4 × 10^5^ at 37 °C) [120]
Phenolic acids (Benzoic acid derivatives)	*p*-hydroxybenzoic acid	No effects were studied (1.0 × 10^3^ at 25 °C) [121]
	Gallic acid	Increase its antioxidant activity (2.0 × 10^3^ at 25 °C) [122]; increase in α-helix content (9.0 × 10^3^ at 25 °C) [79]; transition of α-helix to β-turn structures (1.0 × 10^4^ at 25 °C) [123]
	Ellagic acid	Reduction of α-helix content (1.6 × 10^5^ at 25 °C) [124]
	Vanillic acid	No effects were studied (1.0 × 10^3^ at 25 °C) [121]
	Isovanillic acid	No effects were studied (2.0 × 10^3^ at 25 °C) [122]
	Syringic acid	Increase in antioxidant activity of ligand [122]
	Protocatechuic acid	Increase in antioxidant activity of protocatechuic acid (2.0 × 10^3^ at 25 °C) [122]
	Gentisic acid	Increase in antioxidant activity of gentisic acid (5.0 × 10^3^ at 25 °C) [122]
Phenolic acids (Cinnamic acid derivatives)	Cinnamic acid	Reduction of α-helix content (0.4 × 10^5^ at 25 °C) [125]
	Caffeic acid	Reduction of α-helix content (1.6 × 10^5^ at 25 °C) [125]; stabilization of protein structure (0.3 × 10^5^ at 37 °C) [126]; inhibition of the oxidation of HSA-bound linoleic acid [89]; no effects were studied (0.3 × 10^5^ at 37 °C) [127]; alteration of protein structure (0.2 × 10^5^ at 25 °C) [128]
	Ferulic acid	The partial unfolding of HSA (0.3 × 10^5^ at 25 °C) [129]; binding of ferulic acid inhibits the oxidation of HSA-bound linoleic acid [89]; no effects were studied (2.3 × 10^6^ at 25 °C) [130]
	Sinapic acid	Transition of α-helix to β-turn structures (6.9 × 10^7^ at 25 °C) [123]
	Rosmarinic acid	Changes in tertiary structure with the reduction in α-helix content (0.6 × 10^5^ at 37 °C) [131]; Inhibition of protein glycation and aggregation [132]
	*p*-Coumaric acid	Reduction of α-helix content (1.1 × 10^5^ at 25 °C) [125]
	Chlorogenic acid	The partial unfolding of HSA (0.4 × 10^5^ at 25 °C) [129]; binding of chlorogenic acid inhibits oxidation of HSA-bound linoleic acid [89]; no effects were studied (9.2 × 10^6^ at 25 °C) [130]
Phenolic aldehydes	Vanillin	Reduction of α-helix content (0.6 × 10^5^ at 37 °C) [133]
	Protocatechuic aldehyde	Disordered structure of HSA (9.8 × 10^7^ at 37 °C) [134]
Terpenes (Monoterpenes)	Menthol	Reduction of α-helix content, an increase in β-sheet and random coils [135]
	Cuminaldehyde	Reduction of α-helix content (8.0 × 10^3^ at 25 °C) [136]
	Cuminol	Reduction of α-helix content (1.0 × 10^3^ at 25 °C) [136]
	Saphranal	Reduction of α-helix content, an increase in β-sheet and random coils (3.0 × 10^3^ at 25 °C) [137]
Terpenes (Diterpenes)	Leoheterin	Increase of α-helix content (1.2 × 10^5^ at 25 °C) [138]
	Cafestol	Reduction of α-helix content, increased affinity for warfarin (5.0 × 10^3^ at 25 °C) [139]
	16-*O*-methylcafestol	Reduction of α-helix content, increased affinity for warfarin (8.0 × 10^3^ at 25 °C) [139]
Terpenes (Triterpenes)	Betulinic acid	Reduction of α-helix content, an increase in β-sheet and random coils (1.7 × 10^6^ at 25 °C) [140]
	Asiatic acid	Reduction of α-helix content, an increase in β-sheet and random coils (0.4 × 10^5^ at 25 °C) [141]
	*β*-Carotene	Reduction of α-helix content, an increase in random coils and β-turns (2.7 × 10^5^ at 37 °C) [142]; reduction in α-helix content, an increase in β-turns (3.0 × 10^5^ at 37 °C) [143]
	Isorenieratene	Reduction of α-helix content, increase in β-turns (3.5 × 10^5^ at 37 °C) [143]
Terpenes (Xanthophylls)	Lutein	Reduction of α-helix content, increase in β-turns (3.5 × 10^5^ at 37 °C) [143]
	Astaxanthin	Reduction of α-helix content, an increase in random coils and β-turns (2.6 × 10^6^ at 37 °C) [142]
Apocarotenoids	Crocetin	Reduction of α-helix content, an increase in β-sheet and random coils (2.0 × 10^3^ at 25 °C) [137]
Stilbenes	Resveratrol	Increase of α-helix content, displacement of aflatoxin B1 (6.4 × 10^6^ at 25 °C) [144,145]; protein thermal stabilization [146]
	Rhaponticin	Reduction of α-helix content (1.4 × 10^5^ at 25 °C) [147]
	Esculin	Reduction of α-helix content (4.6 × 10^5^ at 25 °C) [148]
	Esculetin	Reduction of α-helix and increase in β-sheet content (0.3 × 10^5^ at 25 °C) [149]; reduction in α-helix content (0.7 × 10^5^ at 37 °C) [148]
	Scopoletin	Reduction of α-helix content (2.6 × 10^5^ at 25 °C) [86]
	Fraxin	Reduction of α-helix content (3.1 × 10^5^ at 25 °C) [148]
	Fraxetin	Reduction of α-helix content (0.9 × 10^5^ at 25 °C) [148]
	Daphnetin	Reduction of α-helix content (1.7 × 10^6^ at 37 °C) [150]
	Osthole	Conformational change of HSA (1.0 × 10^5^ at 25 °C) [151]; slight reduction in α-helix content, inhibition of HSA esterase activity (8.9 × 10^5^ at 25 °C) [152]
Vitamins	Ascorbic acid	Reduction of α-helix content and increase in β-sheet and random coils (0.2 × 10^5^ at 25 °C) [153]; reduction in α-helix content (0.2 × 10^5^ at 25 °C) [154]; slight changes in secondary structure (3.0 × 10^3^ at 25 °C) [155]
	α-Tocopherol	Reduction of α-helix content and random coils, an increase in β-sheet and β-turn (4.0 × 10^3^ at 25 °C) [153]; impaired binding of diazepam (7.0 × 10^6^ at 25 °C) [156]
	Retinol	Protein stabilization: Increase in α-helix content and reduction in β-sheet content (1.3 × 10^5^ at 25 °C) [157]
Sulfur-containing compounds	Lipoic/Dihydro- lipoic acid	Restoration of zinc ion binding to protein [63]; thermal stabilization of HSA, no effect on trypsin digestion (0.1 × 10^5^ at 37 °C) [56]
Phycobilins	Phycocyanobilin	Displacement of bound bilirubin (2.2 × 10^6^ at 25 °C) [37]; increase in α-helix content and reduction in random coils; increased thermal and proteolytic stability; and conformational change of ligand [49]

**Table 2:** Known interactions between food antioxidants and human transferrin.

Antioxidant Class	Antioxidant Subclass	Chemical Compound	Effects (the Binding Constant in M^−1^, if Available) [Reference]
Flavonoids	Flavonols	Quercetin	Reduction of hydrophobicity in the microenvironment of Trp residue; the unfolding of protein backbone; increase in β-sheet followed by reduction in α-helix and β-turn structures [158]
		Fisetin	Moderate interaction; thermal denaturation of protein (1.4 × 10^6^ at 25 °C) [159]
		Galangin	Reduction of hydrophobicity in the microenvironment of Trp; unfolding of transferrin backbone; increase in β-sheet followed by reduction in α-helix and β-turn structures [158]
		Myricetin	Reduction of hydrophobicity in the microenvironment of Trp; unfolding of transferrin backbone; increase in β-sheet followed by reduction in α-helix and β-turn structures [158]
		Kaempferol	Reduction of hydrophobicity in the microenvironment of Trp; unfolding of transferrin backbone; increase in β-sheet followed by reduction in α-helix and β-turn structures [158]
	Flavones	Luteolin	Increase in hydrophobicity of the Trp microenvironment and thermal stabilization (1.0 × 10^5^ at 35 °C) [160]
		Apigenin	Increase in α-helix content (6.7 × 10^4^ at 25 °C) [161] Increase of hydrophobicity in the Trp microenvironment; thermal stabilization (1.0 × 10^5^ at 35 °C) [160]
		Rutin	Increase of hydrophobicity in the Trp microenvironment; thermal stabilization (2.1 × 10^5^ at 35 °C) [160]
	Isoflavones	Genistein	Slight increase in hydrophobicity in the Trp microenvironment; slight increase in α-helix content (1.3 × 10^4^ at 25 °C) [162]
		Daidzein	Increase of hydrophobicity in the Trp microenvironment; increase in α-helix content (2.9 × 10^5^ at 25 °C) [162]
	Flavanones	Naringenin	Stabilization of transferrin structure; increase in α-helix content; high binding affinity (6.3 × 10^6^ at 25 °C) [161]; high binding affinity; no structural effects were observed [163]
	Anthocyanins	Cyanidin	Changes of hydrophobicity in the microenvironment of Trp and Tyr [164]
Phenolic acids	Cinnamic acid derivatives	Rosmarinic acid	Alteration of protein structure and conformation (4.7 × 10^7^ at 18 °C) [165]
Carotenoids	Xanthophylls	*β*-Cryptoxanthin	No effects were studied [166]
Vitamins		Ascorbic acid	Reduction in α-helix and increase in β-sheet content; inhibition of ligand acid-free radical scavenging activity upon binding (1.1 × 10^4^ at 25 °C) [167]

**Table 3:** Known interactions between food antioxidants and human α2-macroglobulin.

Antioxidant Class	Antioxidant Subclass	Chemical Compound	Effects (the Binding Constant in M^−1^, if Available) [Reference]
Flavonoids	Flavonols	Quercetin	Reduction of hydrophobicity in the Trp microenvironment and α-helix content; reduction in inhibitory activity (4.4 × 10^3^ at 25 °C) [168]
		Myricetin	Increase of hydrophobicity in the Trp and Tyr microenvironment; slight decrease in α-helix content (2.4 × 10^3^ at 45 °C) [169]
Phenolic acids	Benzoic acid derivatives	Gallic acid	Reduction of hydrophobicity in the Trp microenvironment; increase in the α-helix content; reduction in inhibitory activity (2.9 × 10^4^ at 25 °C) [170]
	Cinnamic acid derivatives	Ferulic acid	No significant structural changes were observed (6.7 × 10^4^ at 25 °C) [171]
Vitamins		Ascorbic acid	Induction of slight conformational changes [172]

**Table 4:** Known interactions between food antioxidants and human fibrinogen.

Antioxidant Class	Antioxidant Subclass	Chemical Compound	Effects (the Binding Constant in M^−1^, if Available) [Reference]
Flavonoids	Flavonols	Myricetin	Reduction of α-helix content (2.0 × 10^4^ at 25 °C) [173]
	Flavones	Rutin	No effects were studied (2.1 × 10^4^ at 25 °C) [173]
	Isoflavones	Genistein	Reduction of α-helix content (1.7 × 10^4^ at 25 °C) [173]
		Puerarin	Reduction of α-helix content (8.8 × 10^3^ at 25 °C) [173]
	Flavanols	(-)-Epigallocatechin	No effects were studied [174]
	Flavanones	Hesperidin	Reduction of α-helix content (2.3 × 10^4^ at 25 °C) [173]
		Naringin	Reduction of α-helix content (1.5 × 10^4^ at 25 °C) [173]
Phenolic acids	Cinnamic acid derivatives	Caffeic acid	No effects were studied [175]
		Ferulic acid	No effects were studied [175]
Stilbenes		Resveratrol	No effects were studied [176]; no fibrinogen unfolding/destabilization; mutually protective effect against free radical-induced oxidation (2.6 × 10^3^ at 25 °C) [36]
Tannins	Hydrolyzable tannins	Tannic acid	No effects were studied [176]
Sulfur- containing compounds	Fatty acids	Dehydrolypoic acid	Slight secondary structural alteration, more ordered protein molecular organization; formation of fibrin with thicker fibers; protection from oxidation (1.0 × 10^4^ at 25 °C) [59]

**Table 5:** Known interactions between food antioxidants and human hemoglobin (Hb).

Antioxidant Class	Antioxidant Subclass	Chemical Compound	Effects (the Binding Constant in M^−1^, if Available) [Reference]
Flavonoids	Flavonols	Quercetin	Reduction of α-helix content (1.2 × 10^4^ at 25 °C) [177]
		Dihydromyricetin	No alteration of secondary structure (2.8 × 10^4^ at 23 °C) [178]
	Isoflavones	Genistein	No alteration of secondary structure (3.5 × 10^4^ at 25 °C) [179]
		Diadzein	No alteration of secondary structure (8.7 × 10^3^ at 25 °C) [180]
	Flavanols	Catechin	No alteration of α-helix content; pro-oxidative effect towards metHb formation at higher catechin concentration (7 × 10^7^ at 25 °C) [181]
	Flavanones	Hesperidin	Stabilization of secondary structure (1.4 × 10^4^ at 25 °C) [182] Slight alteration of secondary structure (2.2 × 10^4^ at 25 °C) [183]
		Naringenin	No alteration of secondary structure (1.5 × 10^4^ at 25 °C) [184]
	Anthocyanidins	Procyanidin B3	No effects were studied (0.9 × 10^3^ at 37 °C) [185]
Carotenoids	Terpenes	Astaxanthin	No effects were studied (2.2 × 10^9^ at 37 °C) [185]
	Carotenes	*β*-Carotene	No effects were studied (2.3 × 10^5^ at 37 °C) [185]
Tannins	Hydrolyzable tannins	Tannic acid	Structural changes and protein denaturation [186]; no effects were studied (1.5 × 10^4^ at 25 °C) [187]
Vitamins		L-Ascorbic acid	No effects were studied (4.6 × 10^6^ at 37 °C) [185]
	Tocopherols	*α*-Tocopherol	No effects were studied (3.2 × 10^3^ at 37 °C) [185]
Other	Turmeric	Curcumin	Unfolding of protein; reduction in thermal stability (4.9 × 10^5^ at 20 °C) [188]

## 5. Redox Status, Antioxidants, and Diseases

Oxidative stress underlines numerous chronic and degenerative impairments and participates in aging, apoptosis, cancer, pathogen infection, diabetes, cardiovascular, neurological, and other diseases. A human organism has its defense mechanisms, but that capacity is often overcome, and exogenous antioxidants from food are needed to support the combat. Redox equilibrium in the body plays a critical role in health maintenance, especially in scavenging free radicals and free metal ions and modifying cell signaling, host immunity, and gene expression [189].

Many antioxidants have been recognized to exert beneficial health effects [8]. Although it is generally accepted that consuming food rich in antioxidants protects against cancer, there is no sufficient evidence to support their therapeutic impact on patients. Likewise, our knowledge of the combined effect of regular therapy assisted by antioxidants is limited. Antioxidants are believed to alleviate the side effects of cancer therapy and increase tolerance for treatment [190]. On the other hand, antioxidants can interfere with chemo- or radiotherapy, reducing the efficiency of the treatment. Even more, some data suggest the assistant role of antioxidants when cancer transformation has already started or an increased risk of cancer recurrence [191].

Antioxidants protect neurons against oxidation, particularly lipid components of cell membranes contributing to cell integrity and proper neurotransmission. Some neurodegenerative disorders, such as Alzheimer’s disease, are accompanied by the formation of protein aggregates; oxidative stress favors protein modification and cross-linking into insoluble fibrils [192]. Food antioxidants have also improved diabetic complications, such as insulin resistance and impaired insulin response [193]. Increased ROS production, impaired mitochondrial antioxidant defense, altered function of endothelial cells, and the influx of calcium ions are involved in cardiovascular diseases such as atherosclerosis, hypertension, and ischemic heart disease. Several food antioxidants have been documented to reduce the risk of cardiovascular diseases, most often associated with the consumption of leafy greens, citrus fruits, red wine, chocolate, tea, and coffee. Food antioxidants not digested in the stomach pass into the colon and contribute to maintaining gut microbiota by stimulating the growth of beneficial bacterial species (such as *Lactobacillus*, *Enterococci*, and *Bifidobacterium* species) and inhibiting pathogens (such as *Clostridium*). However, each antioxidant exerts a positive/negative effect on a specific set of bacteria [194,195]. The exact mechanisms of most antioxidant-assisted health effects have yet to be clearly defined.

## 6. Future Perspectives in the Analysis of Antioxidant/Protein Interactions

Understanding interactions between antioxidants and proteins gained increased attention in the last two decades, although most investigations still employ in vitro experiments. Relatively simple models are needed to capture the effect of a particular antioxidant binding to a specific protein. Most research work has been performed using one protein and one antioxidant. Examination of the binding event of several antioxidants and one protein was rarely conducted. Such investigation, for example, includes follow-up of the effects when the whole plant extracts are assayed, as mentioned previously. Examination of the binding effect of a particular antioxidant with several proteins is an even rarer study design. It was employed only with simple systems, not having too many protein species. For example, phenolic compounds were isolated from the albumin, glutelin-1, glutelin-2, prolamin, and globulin wheat protein fractions and were identified by HPLC/LC-MS [196]. The binding of polyphenols to caseins, BLG, albumin, and lactoferrin in the commercial bovine skimmed milk powder was also traced [197]. Similar studies were not found for human physiological samples. Some experiments, mostly with animal proteins, were performed to investigate biological effects on cells in culture. For example, protein-bound curcumin expresses superior anti-cancer activity on human breast carcinoma and neuroblastoma cell lines compared to free curcumin [198], suggesting that antioxidant/protein complexes may become a novel tool in treating diseases. More work on cell cultures is undoubtedly needed to document the overall effect of antioxidant/protein complexes on the living unit, taking into account its growth and metabolic activity.

Antioxidants behave according to their structure and nature, and their antioxidant capacity, when bound to proteins, may or may not be affected, depending on the structure and nature of proteins. It is also worth mentioning that analytical conditions in assays employed for in vitro measurement of an antioxidant capacity are not the same as conditions in vivo, suggesting caution in extrapolating in vitro results to living beings. There are hundreds of antioxidant derivatives in food, many more metabolites, and only a fraction have been studied. Likewise, few proteins were assayed as antioxidant-binding partners and very few were human proteins. According to the Plasma Proteome Database, more than 10.000 plasma proteins exist, or several billion in the entire human proteome; most have yet to be quantified due to very low concentrations [199]. They are all potential partners for the interaction with antioxidants.

Most articles report that the interaction was recorded, evaluated as a structural alteration of the protein, and sometimes characterized as a change in the function of the protein or antioxidant. As mentioned, some results do not agree between studies and may be conflicting. Future work should be directed into a deeper analysis of the already known complexes and search for the new associations and their examination in relation to cell/extracellular milieu defined by specific environmental features and molecular species, such as pH, free radicals, redox-reactive small molecules, redox enzymes, etc. Antioxidant/protein complexes should also be examined concerning food habits and (patho)physiological conditions. It is certainly intriguing to learn about mechanisms that govern and regulate the distribution of many antioxidants between many proteins in the body, and not only between proteins but other molecules as well. Methodological approaches for these investigations are, however, limited. Separation of individual proteins from a complex mixture, such as human plasma, without disturbing antioxidant/protein interaction and detection of bound individual antioxidants is still an issue to resolve. Improvements in the existing analytical methods and the developing of more sensitive and specific techniques to handle that task are needed.

## 7. Conclusions

Some proteins bind antioxidants, and specific ligands only bind to some proteins. Some antioxidants are present in forms that may directly bind to proteins, some after processing, and some are quickly transformed into derivatives with characteristics different from parent molecules. Some antioxidants compete, and some act in synergy. Only some protein functions are affected by antioxidant binding, and different antioxidants exert different effects. There are hundreds of food antioxidants and thousands of plasma proteins, which may form a coupling pair. Finally, there are severe methodological limitations of analytical techniques that can capture antioxidant/protein interactions that occur in a complex system such as the human body and regarding interferences from other biochemical processes. Having in mind all said, the investigation of antioxidant/protein interactions at the level of the human organism, the determination of antioxidant distribution between proteins, and involvement in the particular physiological role is a very complex and challenging task. However, by knowing the role of a particular protein in certain pathology or aging, and the effect exerted by a particular antioxidant bound to it, it is possible to recommend specific food intake or resistance to it to improve the condition or slow down the process.

## Data Availability

Not applicable.
